# Evaluation of vector competence of *Culex tritaeniorhynchus* and *Culex pipiens pallens* for Japanese encephalitis virus genotype III and V

**DOI:** 10.1186/s13071-025-07180-5

**Published:** 2025-12-05

**Authors:** Ji-Young Kwon, Hyun Hee Jung, Hee Il Lee, Bo-Ram Yun

**Affiliations:** Division of Vectors and Parasitic Diseases, Korea Disease Control and Prevention Agency, 187, Osongsaengmyeong 2-Ro, Osong-Eup, Heungdeok-Gu, Cheongju-Si, Chungcheongbuk-Do Republic of Korea

**Keywords:** Japanese encephalitis, Mosquito-Borne diseases, *Culex*, Mosquito vectors, Genotype

## Abstract

**Background:**

Japanese encephalitis virus (JEV) is a major mosquito-borne pathogen, primarily transmitted by *Culex tritaeniorhynchus* in rural regions. In the Republic of Korea (ROK), genotype V (GV) has become the dominant JEV strain since 2010, raising suspicion about the vector competence of urban mosquitoes like *Culex pipiens pallens*.

**Methods:**

This study evaluated the vector competence of *Cx. tritaeniorhynchus* and *Cx. pipiens pallens* for JEV GIII and GV under laboratory conditions. Mosquitoes were orally infected, and the infection rate (IR), dissemination rate (DR), and transmission rate (TR) were assessed at days 7 and 14 post-infection.

**Results:**

*Culex tritaeniorhynchus* showed consistently high IR, DR, and TR for both genotypes, with over 95% of mosquitoes infected and actively transmitting the virus. In contrast, *Cx. pipiens pallens* exhibited a markedly lower IR, ranging from 23.1 to 39.2%; however, among infected mosquitoes, DR and TR were comparatively high. Viral load and titers were also markedly higher in *Cx. tritaeniorhynchus* than in *Cx. pipiens pallens*, particularly in the head-thorax and salivation samples.

**Conclusions:**

These findings confirm that *Cx. tritaeniorhynchus* is a highly competent vector for JEV GIII and GV and suggest that *Cx. pipiens pallens* may play a notable role in the transmission of Japanese encephalitis virus in urban areas. This study emphasizes the importance of targeted vector surveillance and control strategies for multiple mosquito species, especially given the recent urbanization of JE cases in the ROK.

**Graphical Abstract:**

**Figure:**
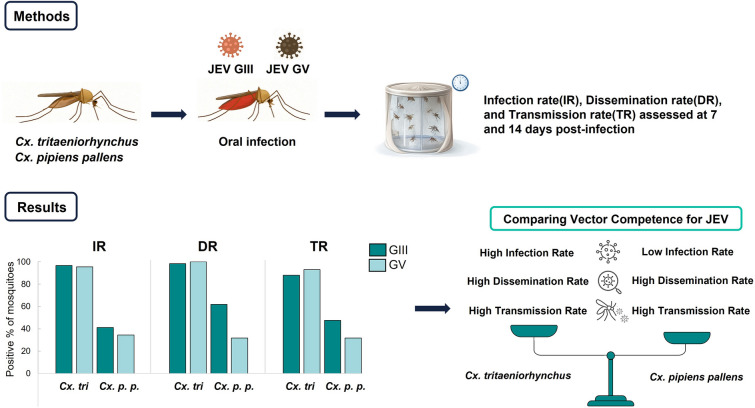

## Background

Japanese encephalitis virus (JEV) is a major mosquito-borne zoonotic disease first reported in the late 1800s and initially isolated in Japan in 1933 [1]. JEV is a leading cause of viral encephalitis in many Asian countries, with approximately 100,000 clinical cases reported annually [2]. JEV infection can affect individuals of all age groups; however, it primarily affects children under the age of 14 in endemic regions [1, 3]. JEV infection is generally mild or asymptomatic. However, approximately 1 in 250 cases may progress to severe disease, characterized by high fever, headache, neck stiffness, disorientation, coma, seizures, spastic paralysis, and risk of mortality. Among symptomatic individuals, the case fatality rate can reach 30%. Additionally, 20–30% of survivors experience permanent cognitive, behavioral, or neurological sequelae [2]. JEV is primarily transmitted by culicine mosquitoes and maintained in an enzootic cycle involving wading birds and pigs as amplifying hosts, whereas humans serve as incidental and dead-end hosts [4, 5].

JEV belongs to the genus *Orthoflavivirus* within the family Flaviviridae. It is an enveloped, single-stranded, positive-sense RNA virus [6, 7]. JEV consists of three structural proteins (C, M, and E) and seven non-structural proteins (NS1, NS2A, NS2B, NS3, NS4A, NS4B, and NS5) [8]. Based on the sequence of the E gene, it can be phylogenetically classified into five distinct genotypes (GI to GV) [8, 9]. At the molecular level, substantial differences have been observed between GV and other genotypes. Before the 1990s, GIII was predominant; however, since then, GI infections have markedly increased worldwide [10]. Currently, GIV and GV are associated with localized outbreaks [10]. In 2022, GIV was reported for the first time in Australia, while GV has been circulating in both China and the Republic of Korea (ROK) since 2009 [11–13]. Particularly in the ROK, GV has been consistently detected since 2010, suggesting that it has become the dominant strain in the region [10, 13].

*Culex tritaeniorhynchus* is considered the primary vector of JEV in the endemic regions of Asia, including Japan and the ROK, whereas *Culex pipiens* complex is typically not regarded as a primary vector [14–16]. However, given the high prevalence and adaptability of JEV in temperate regions, *Cx. pipiens* could play a crucial role as a potential vector if JEV were introduced into new areas [17, 18]. Under laboratory conditions, the vector competence of *Cx. tritaeniorhynchus* was evaluated for GI, GIII, and GV in Japan [14]. Similarly, vector competence of *Cx. pipiens* complex has been demonstrated for GIII in Switzerland and the UK [12, 19] and evaluated for both GI and GIII in China, as well as for GIII and GV in France [18, 20]. While most previous studies have focused on JEV GIII, GV has been repeatedly detected in *Cx. pipiens* complex in the ROK. However, no studies have yet evaluated the vector competence of this species for JEV in the ROK, highlighting the importance of the present investigation.

From 2020 to 2023, 58 cases of Japanese encephalitis (JE) were reported in the ROK, including nine fatalities [21]. JE cases were primarily reported in rural areas such as Jeollanam-do and Chungcheongnam-do during the 1980s. However, recent cases have been reported in Gyeonggi-do (23 cases), Seoul (8 cases), and Gangwon-do (6 cases) [21, 22]. This shift may be associated with increasing urbanization, climate change, or ecological adaptation of mosquito vectors to urban environments. Although most current JE cases now occur in urban areas*, Cx. tritaeniorhynchus* is still dominant in rural habitats. This suggests the possible involvement of alternative mosquito species, such as *Cx. pipiens pallens*, in the transmission of JEV in urban areas.

This study experimentally evaluated the vector competence of *Cx. tritaeniorhynchus*, a mosquito species primarily found in rural areas, and *Cx. pipiens pallens*, which is more commonly found in urban settings, for JEV GIII and GV.

## Methods

### Mosquitoes

Laboratory colonies of *Cx. tritaeniorhynchus* (F14) and *Cx. pipiens pallens* (F32) were used in the infection experiments. Those two species were originally collected from Hongseong (in 2020) and Cheongju (in 2019), ROK, respectively. *Culex pipiens pallens* had been previously confirmed by PCR amplification of the acetylcholinesterase-2 (ace-2) gene, following the protocol described by Smith and Fonseca [23]. Rearing was carried out at 27 ± 1 °C, 70 ± 5% relative humidity, and under a 12 L:12 D photoperiod (12 h of light and 12 h of darkness). Adults were provided with a 10% sucrose solution, and females were collected weekly by artificial aspiration for experiment of JEV infection.

### Virus production and titration

JEV GIII was isolated from *Cx. tritaeniorhynchus* in 1994 [24], whereas GV was isolated from *Culex orientalis* in 2020 (Table 1) [25]. We propagated the viruses in Vero cells as previously described [25, 26]. The viruses were cultured in Eagle’s Minimal Essential Medium (MEM, WELGENE, ROK) supplemented with 10% fetal bovine serum (FBS, Thermo Fisher Scientific, Waltham, MA, USA), 100 U/ml penicillin, and 100 μg/ml streptomycin (Gibco, Thermo Fisher Scientific, Waltham, MA, USA) at 37 °C in a 5% CO₂ incubator for 3 days in T75 flasks. To confirm viral replication, we evaluated the cytopathic effects (CPE) using an optical microscope. BHK-21 cells were seeded into six-well plates 1 day prior to virus infection [25]. The virus was serially diluted tenfold in MEM supplemented with 2% FBS and 1% penicillin/streptomycin (P/S). Subsequently, 300 µl of each dilution was inoculated into individual wells. The plates were incubated at 37 °C with 5% CO₂ for 1 h. Following incubation, the inoculum was replaced with 4 ml of overlay medium containing 0.5% agarose, 2% FBS, and 1% P/S in MEM. We further incubated the plates at 37 °C with 5% CO₂ for 5 days to allow plaque formation. The cells were fixed with 4% paraformaldehyde for 30 min and stained with 1% crystal violet. The number of plaques was counted, and the viral titer was calculated as plaque-forming units per milliliter (PFU/ml).

**Table 1:** Japanese encephalitis virus (JEV) genotypes (GIII and GV) used in this study

JEV genotype	Year	Source	Virus titer (PFU/ml)	Accession no.	References
GIII	1994	*Culex tritaeniorhynchus*	1.2 × 10^6^–1.0 × 10^7^	FJ938217	Yun et al. [24]
GV	2020	*Culex orientalis*	2.0 × 10^6^–1.0 × 10^7^	NP478074	Seo et al. [25]

PFU, plaque-forming units

### Oral infection of mosquitoes

Oral infections of mosquitoes were conducted at the BSL-3 Laboratory of the Division of Vectors and Parasitic Diseases (Korea Diseases Control and Prevention Agency, KDCA). Female mosquitoes aged 5–7 days were deprived of sucrose for 24 h before providing the infectious blood meal. Subsequently, we fed the mosquitoes the infectious blood using a Hemotek membrane feeding apparatus (Hemotek Ltd., Accrington, Lancashire, UK) in a dark environment maintained at 27 ± 1 °C overnight. The infectious blood meal consisted of a mixture of defibrinated sheep blood (MBcell, Seoul, ROK) and virus in a 2:1 ratio, with ATP (final concentration: 10 nM) added as a phagostimulant. The viral titer in the blood meal was adjusted to 1.2 × 10^6^ to 1.0 × 10^7^ PFU/ml for GIII and 1.0 × 10^6^ to 1.0 × 10^7^ PFU/ml for GV. Blood-fed female mosquitoes were sorted and reared at 27 ± 1 °C with a relative humidity of 70 ± 5% under a 12L:12D photoperiod.

### Mosquito dissections and salivation

To confirm viral infection, we dissected the mosquitoes on days 7 and 14 post-infection (dpi). For each mosquito, the midgut, legs and wings, and head-thorax were separated and collected into individual tubes containing 300 μl of MEM supplemented with 2% FBS and a homogenization bead, to assess infection, dissemination, and transmission, respectively.

At 14 dpi, additional transmission analysis was conducted by collecting saliva. The legs and wings of cold-anesthetized mosquitoes were removed, and each proboscis was inserted into a 10-μl droplet of phosphate-buffered saline (PBS) held within a cut pipette tip for 30 min. All body parts and saliva samples were stored at –80 °C until further analysis.

### RNA extraction and JEV detection

Except for saliva samples, all collected tissues were homogenized prior to RNA extraction by two cycles of bead beating at 7500 rpm for 30 s each. We then centrifuged the homogenates at 13,000 rpm for 1 min. To measure the viral titer, we transferred 30 μl of the supernatant from each homogenized sample to a new tube. Total RNA was extracted using the Clear-S™ Total RNA extraction kit (Invirustech, Gwangju, Republic of Korea), following the manufacturer’s instructions. To detect the NS5 gene of flavivirus, qRT-PCR was performed with a Clear-MD® flavivirus real-time RT-PCR detection kit (Invirustech, Gwangju, Republic of Korea). The qRT-PCR conditions for each reaction were as follows: cDNA synthesis at 45 °C for 10 min, reverse transcriptase inactivation at 95 °C for 10 min, 40 cycles of denaturation at 95 °C for 10 s, annealing at 60 °C for 15 s, and extension at 72 °C for 10 s, followed by signal reading at 80 °C for 15 s. After amplification, the qRT-PCR products were subjected to a melting curve analysis to verify each product by its specific melting temperature: denaturation at 95 °C for 30 s, annealing at 70 °C for 30 s, and a gradual temperature increase (0.5 °C increments) to 95 °C in 30 s. qRT-PCR reactions were analyzed by cycle threshold (Ct) values, with Ct ≤ 40 interpreted as positive for JEV RNA. Melting curve analysis was performed with the manufacturer’s instructions; samples with peaks at 82–88 °C were considered positive (positive control 86–89 °C).

### JEV titration

We prepared samples collected from legs-wings, body, and head-thorax on days 0, 7, and 14 dpi, using 30 µl of homogenate per sample. The viral titer was determined as 50% tissue culture infectious dose per milliliter (TCID_50_/ml) using BHK-21 cells. For viral titration, BHK-21 cells were seeded into 96-well cell culture plates at a density of 1.0 × 10^4^ cells/well in 200 µl of MEM supplemented with 5% FBS and 1% P/S. Serial tenfold dilutions of the virus were prepared in MEM containing 2% FBS. Subsequently, 100 µl of each virus dilution was added to five replicate wells, which were then incubated at 37 °C for 5 days. On the fifth day, the cells were fixed and stained with crystal violet to visually evaluate the CPE. The TCID_50_/ml was calculated using the Reed-Muench method, based on the number of wells exhibiting CPE at each dilution [27].

### Statistical analysis

Statistical analyses were performed using GraphPad Prism software version 5.01 (GraphPad Software, San Diego, CA, USA). Differences in infection rate (IR), dissemination rate (DR), and transmission rate (TR) between JEV genotypes were evaluated using two-tailed Fisher’s exact test. When multiple pairwise comparisons were conducted, Bonferroni correction was applied, and the results were denoted by letter coding. To compare viral RNA loads among mosquito tissues, the non-parametric Kruskal-Wallis test was applied because of the non-normal distribution of the data, followed by Dunn’s multiple comparison test with Bonferroni correction for pairwise comparisons. Population transmission rates (PTR) between time points were compared using t-test. Statistical significance was set at *p* < 0.05.

## Results

### JEV infection rates (IR), dissemination rates (DR), and transmission rates (TR)

Infection, dissemination, and transmission rates of *Cx. tritaeniorhynchus* and *Cx. pipiens pallens* were evaluated at 7 and 14 dpi for JEV GIII and GV (Table 2). *Culex tritaeniorhynchus* consistently exhibited high vector competence for both genotypes, with IR, DR, and TR exceeding 95% across time points. In contrast, *Cx. pipiens pallens* showed lower and more variable responses. For GIII, IR remained moderate across time (39.2–41.2%), with corresponding increases in DR and TR at 14 dpi. GV-infected *Cx. pipiens pallens* exhibited even lower IR. Although high DR and TR were observed at 7 dpi, both declined markedly by 14 dpi.

**Table 2:** Comparisons of the infection (IR), dissemination (DR), and transmission rates (TR) of *Culex tritaeniorhynchus* and *Culex pipiens pallens* infected with two different genotypes (GIII and GV) of Japanese encephalitis virus (JEV) after artificial infection

	7 dpi			14 dpi			
	IR	DR	TR	IR	DR	TR	TR (salivation)
*Cx*. *tritaeniorhynchus*							
JEV GIII	56/57 (98.2%)^a^	56/56 (100.0%)^a^	55/56 (98.2%)^a^	58/60 (96.7%)^a^	57/58 (98.3%)^a^	58/58 (100.0%)^a^	51/58 (87.9%)^a^
JEV GV	53/53 (100.0%)^a^	47/53 (88.7%)^a^	51/53 (96.2%)^a^	42/44(95.5%)^a^	42/42 (100.0%)^a^	42/42 (100.0%)^a^	39/42 (92.9%)^a^
*Cx. pipiens pallens*							
JEV GIII	20/51 (39.2%)^b^	7/20 (35.0%)^b^	5/20 (25.0%)^b^	21/51 (41.2%)^b^	13/21 (61.9%)^a,b^	13/21 (61.9%)^a,b^	10/21 (47.6%)^b^
JEV GV	12/52(23.1%)^b^	10/12 (83.3%)^a,b^	8/12 (66.7%)^a,b^	22/64 (34.4%)^b^	7/22 (31.8%)^b^	8/22 (36.4%)^b^	7/22 (31.8%)^b^

The following formulas were used to calculate vector competence parameters

Infection rate (IR) = the number of JEV-positive bodies/total number of surviving blood-fed females

Dissemination rate (DR) = the number of JEV-positive legs and wings samples/the number of JEV-positive bodies

Transmission rate (TR) = the number of JEV-positive head, thorax, or saliva samples/the number of JEV-positive bodies

Statistical comparisons of IR, DR, and TR between JEV genotypes (GIII vs. GV) were performed separately for each mosquito species and time point using Fisher’s exact test with Bonferroni correction. Different lowercase letters (a, b) within the same column indicate statistically significant differences between genotypes (*p* < 0.05), whereas values sharing the same letter are not significantly different (*p* > 0.05). Dpi, days post-infection

### Quantification of viral RNA in mosquito tissues

**Fig. 1:**
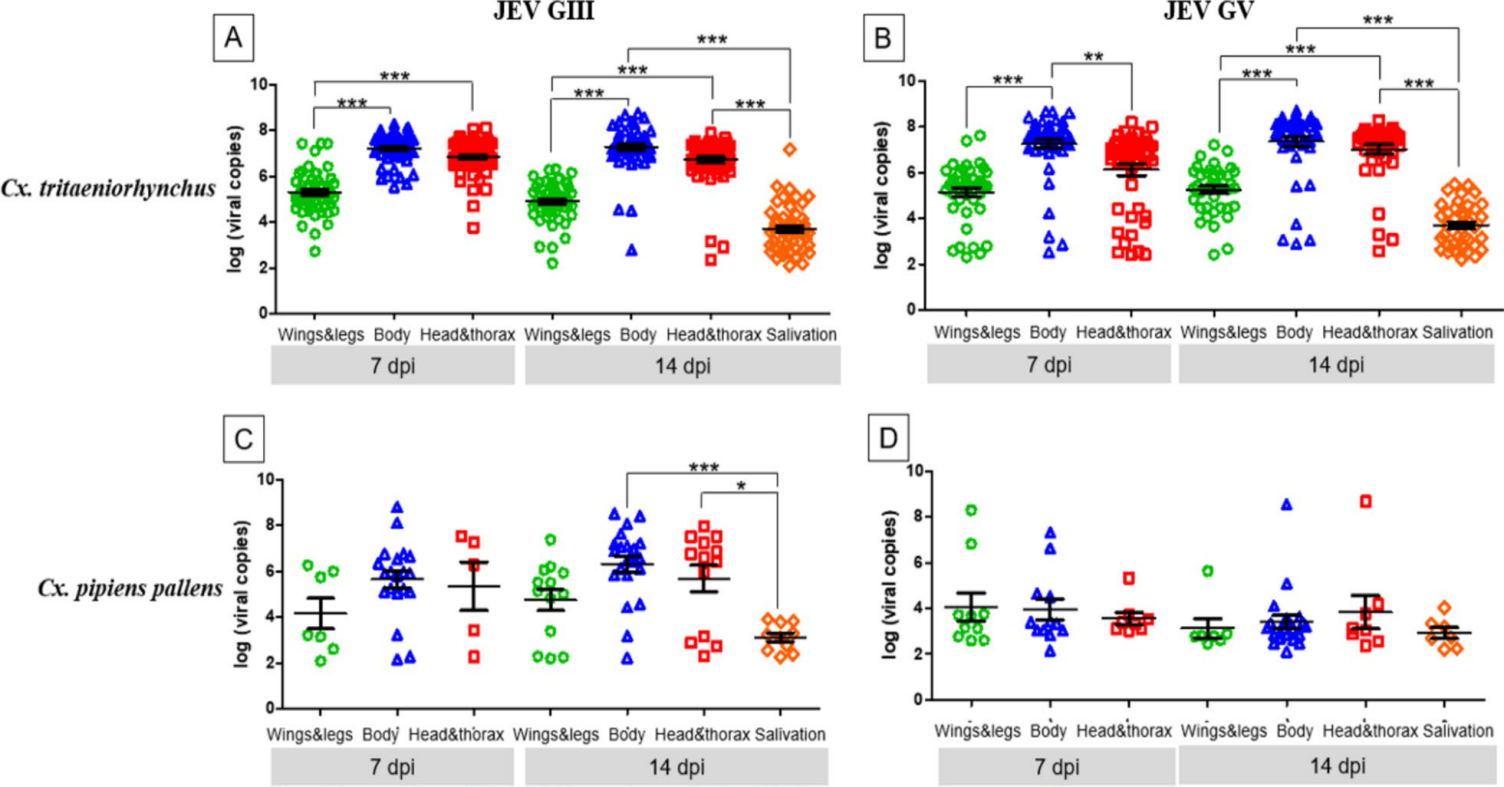
Differences in viral RNA loads of *Culex tritaeniorhynchus* (A, B) and *Culex pipiens pallens* (C, D) infected with two different genotypes (GIII and GV) of Japanese encephalitis virus (JEV)

Quantitative analysis of viral RNA loads revealed distinct replication and dissemination patterns among mosquito species and JEV genotypes (Fig. 1). In *Cx. tritaeniorhynchus* infected with GIII, viral RNA loads differed significantly among tissues at both 7 and 14 dpi (Kruskal-Wallis H = 254.06, df = 3, *p* < 0.0001). Dunn’s multiple comparison test showed that RNA levels in the body and head-thorax were significantly higher than those in the legs-wings at both time points (all *p* < 0.001). At 14 dpi, viral RNA levels in salivation samples were further elevated relative to other tissues (*p* < 0.001), indicating active dissemination and transmission competence at this stage. Similarly, in *Cx. tritaeniorhynchus* infected with GV, viral RNA loads also differed significantly among tissues at 7 and 14 dpi (Kruskal-Wallis H = 161.03, df = 3, *p* < 0.0001). At 14 dpi, the highest viral accumulation was detected in salivation samples (*p* < 0.001), suggesting efficient viral dissemination and enhanced transmission potential at infection stages. In *Cx. pipiens pallens* infected with JEV GIII, viral RNA loads also varied significantly among tissues (Kruskal-Wallis H = 22.95, df = 3, *p* < 0.001). RNA levels were significantly higher in the body and head-thorax than in the salivation samples at 14 dpi (*p* < 0.001 and *p* < 0.05, respectively), whereas no significant differences were observed at 7 dpi. By contrast, RNA loads did not differ significantly among tissues or time points in *Cx. pipiens pallens* infected with JEV GV (Kruskal-Wallis H = 7.83, df = 3, *p* = 0.25). These results demonstrate that *Cx. tritaeniorhynchus* supports robust replication and dissemination of both JEV genotypes—particularly evident in salivation at 14 dpi—confirming its high transmission potential.

Viral RNA loads were determined by RT-qPCR targeting the JEV NS5 gene, representing relative viral RNA copies in each body part. The analyzed body parts include legs-wings (green), body (blue), head-thorax (red), and salivation (orange). Viral loads are expressed as log₁₀ (viral copies). Bars represent the mean ± standard error of the mean (SEM).

Differences in viral RNA loads among mosquito body parts were analyzed using the Kruskal-Wallis test, followed by Dunn’s multiple comparison test with Bonferroni correction for pairwise comparisons. Statistical significance levels are indicated as follows: ***p < 0.001, **p < 0.01, *p < 0.05.

### Infectious viral titers

Infectious viral titers (log TCID₅₀/ml) were generally consistent with the RNA load patterns observed across tissues (Table 3). In *Cx. tritaeniorhynchus*, viral titers were stable or slightly increased over time for both JEV genotypes. Notably, GV showed a significant increase in head-thorax viral titers between 7 and 14 dpi (t-test, *p* = 0.0126), whereas GIII showed no significant difference (*p* > 0.05). In *Cx. pipiens pallens*, GIII titers increased slightly over time in the head-thorax but not statistically significantly (*p* = 0.366). GV-infected mosquitoes exhibited consistently low infectious titers throughout the experimental period with no significant temporal variation (*p* > 0.05). Overall, these results indicate that infectious viral titers in *Cx. tritaeniorhynchus* particularly for GV were significantly elevated at later infection stages, whereas *Cx. pipiens pallens* maintained low viral titers, which were statistically non-significant, over time, reflecting its comparatively lower replication efficiency.

**Table 3:** Mean viral titers (log TCID₅₀/ml) of two Japanese encephalitis virus (JEV) genotypes (GIII and GV) in infected *Culex tritaeniorhynchus* and *Culex pipiens pallens*

Source	Dpi	Mean viral titers (log TCID₅₀/ml)	
		GIII	GV
*Cx*. *tritaeniorhynchus*			
Legs-wings	7	1.27 ± 0.078	1.56 ± 0.073
	14	1.68 ± 0.041	1.45 ± 0.087
Body	7	2.03 ± 0.060	2.95 ± 0.093
	14	2.13 ± 0.091	3.06 ±0.095
Head-thorax	7	1.63 ± 0.130	2.36 ± 0.112
	14	2.48 ± 0.099	2.81 ± 0.092
*Cx. pipiens pallens*			
Legs-wings	7	1.88 ± 0.022	1.35 ± 0.217
	14	1.52 ± 0.045	1.62 ± 0.050
Body	7	2.01 ± 0.093	1.01 ± 0.092
	14	2.40 ± 0.089	1.16 ± 0.175
Head-thorax	7	1.97 ± 0.120	0.46 ± 0.042
	14	2.57 ± 0.061	1.55 ± 0.180

Viral titers were determined by TCID₅₀ assays on BHK-21 cells and expressed as log TCID₅₀/ml. Each value represents the mean ± SEM from three independent tissue pools.

These data represent infectious viral titers across mosquito tissues and complement the RT-qPCR results shown in Fig. 1.

### Population transmission rates

**Fig. 2:**
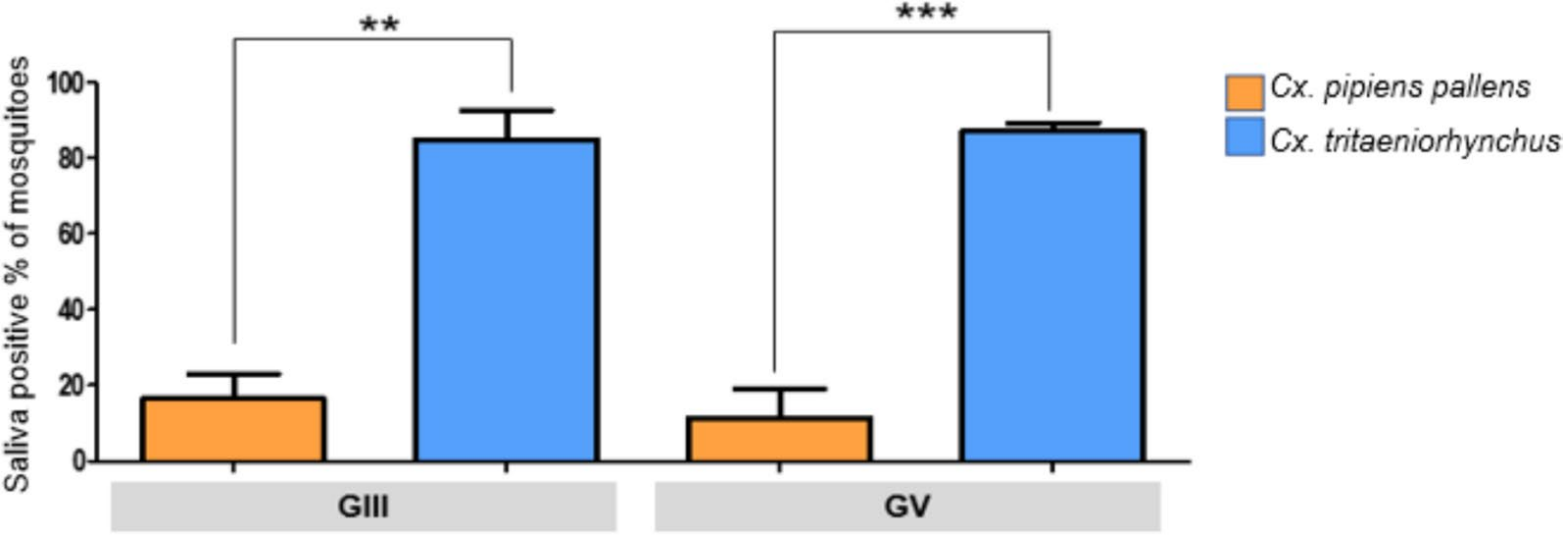
Comparison of population transmission rates (PTR) between *Culex pipiens pallens* and *Culex tritaeniorhynchus* for Japanese encephalitis virus (JEV) genotype III and V at 14 dpi. PTR is defined as the proportion of JEV-positive saliva out of the total number of surviving blood-fed female mosquitoes at 14 dpi. Significant differences between species are denoted as follows: **p < 0.01, ***p < 0.001. Error bars indicate standard error of the mean (SEM)

The PTR, calculated as the proportion of saliva-positive mosquitoes among all blood-fed individuals, served as an indicator of transmission potential. At 14 dpi, PTR of two species showed significant difference. For GIII, *Cx. tritaeniorhynchus* maintained a high PTR of approximately 85%, while *Cx. pipiens pallens* demonstrated a slight increase to around 16% (*p* = 0.0024). In GV, *Cx. tritaeniorhynchus* reached a PTR of nearly 87%, while *Cx. pipiens pallens* remained < 11% (*p* = 0.0001). These findings indicate that *Cx. tritaeniorhynchus* is a more efficient vector for both JEV GIII and V than *Cx. pipiens pallens*, highlighting its critical role in the epidemiology of JEV transmission (Fig. 2).

## Discussion

JEV GV was first isolated from a human patient in Malaysia in 1952 but then was not reported again for nearly 5 decades [13]. Since its re-emergence, GV has been detected in mosquito populations including *Cx. tritaeniorhynchus* in China (2009) and *Culex bitaeniorhynchus* in the ROK (2010), and later in *Cx. orientalis* and the *Cx. pipiens* complex in the ROK (2012) [13, 28, 29]. The first confirmed human case of JEV GV in the ROK occurred in 2015 [30]. Although *Cx. tritaeniorhynchus* is recognized as the primary vector of JEV in endemic regions of Asia, JEV GV has not yet been detected in ROK.

In this study, we evaluated the vector competence of *Cx. tritaeniorhynchus* and *Cx. pipiens pallens* for JEV genotypes GIII and GV under laboratory conditions. As expected, *Cx. tritaeniorhynchus* exhibited high susceptibility to both genotypes, with IR, DR, and TR rates exceeding 87% and consistently high viral loads in saliva. These findings align with previous reports, highlighting the high competence of this species for JEV genotypes GI, GIII, and GV [14, 31].

In *Cx. pipiens pallens*, GV-infected mosquitoes at 7 dpi showed a low IR (23.1%) but unexpectedly high DR (83.3%) and TR (66.7%). This pattern reflects interpretative limitations owing to the small number of infected specimens at this early time point. In contrast, GIII-infected mosquitoes at the same time point exhibited a higher IR (39.2%) but lower DR and TR (35.0% and 25.0%, respectively), suggesting slower viral progression. At 14 dpi, GIII showed increased DR and TR (both 61.9%), whereas GV rates declined. These findings indicate that the vector competence of *Cx. pipiens pallens* varies depending on the viral genotype. This pattern suggests that viral replication and transmission within mosquitoes vary depending on both mosquito species and viral genotypes. Prior studies have reported substantial geographic variability in the competence of the *Cx. pipiens* complex, likely driven by differences in mosquito origin, viral genotype, and environmental conditions such as ambient temperature [5, 18, 20]. For instance, *Cx. pipiens* in Sweden showed an infection rate of only 12% for JEV GIII, whereas populations in China achieved rates above 40% under warmer conditions [5, 18]. These findings suggest that environmental and biological factors, including genotype-vector compatibility and local adaptation, may modulate the potential of *Cx. pipiens* complex as a secondary vector of JEV.

Epidemiological patterns in the ROK support this possibility. Since 2010, a substantial proportion of JE cases have occurred in urbanized northern regions such as Seoul and Gyeonggi-do, despite the predominance of *Cx. tritaeniorhynchus* in southern regions and rural habitats such as rice paddies and livestock farms [30, 32]. The prevalence of *Cx. pipiens* complex in urban areas, coupled with rising urban JE cases, suggests that alternative transmission cycles may be operating in these environments. In the past, JE cases have also been reported in regions where *Cx. tritaeniorhynchus* had very low densities, such as Gangwon-do (ROK, 2010) and Hokkaido (Japan, 1989), raising concerns at the time about the possible involvement of other mosquito species in JEV transmission [28, 33, 34].

Several urban-adapted mosquito species, including *Culex pipiens pallens*, *Cx. quinquefasciatus*, *Cx. modestus ficalbi*, *Cx. orientalis, Cx. bitaeniorhynchus*, and *Armigeres subalbatus*, have been proposed as potential or secondary vectors of JEV, with vectorial roles that may vary depending on ecological and geographical conditions [14, 20, 26, 35]. Among them, the *Cx. pipiens* complex is of particular interest because of its opportunistic host-feeding behavior. Although *Cx. tritaeniorhynchus* has been considered a key vector in the classical pig-mosquito-human transmission cycle, its tendency to feed on a range of nonhuman mammalian hosts, including pigs and cattle, may influence transmission dynamics depending on host community composition [36, 37]. In contrast, *Cx. pipiens* often feeds on birds and humans, facilitating potential alternative cycles in urban areas [37, 38]. Supporting this, human JE cases have been documented in areas where pig farming is absent or has been discontinued, including metropolitan Seoul and Singapore [22, 39]. Moreover, domestic birds and wild boars have been shown to develop sufficient viremia to infect mosquitoes, further supporting their role as possible amplification hosts [22, 40, 41]. These findings suggest that the conventional transmission model may not fully account for JE epidemiology in changing landscapes, particularly under conditions of urbanization and ecological disruption.

Although our study focused on GIII and GV, the high genetic similarity between GI and GIII, along with previous evidence, suggests that both *Cx. tritaeniorhynchus* and *Cx. pipiens pallens* may also be competent for GI [18, 42]. However, this hypothesis remains to be experimentally validated. Given the recent dominance of GV in the ROK and the potential for future genotype shifts, further studies evaluating a broader range of mosquito species with viral genotypes are needed.

This study had several limitations. The laboratory experiments did not fully replicate natural conditions, and only a single mosquito colony and viral strain were used for experiments. In addition, the GIII strain (K94A071) employed in this study was not a recent field isolate but an archived Korean reference strain. Therefore, genetic or phenotypic differences that may have arisen in more recent GIII isolates could not be assessed. One limitation of this study is that viral infection and transmission were evaluated only up to 14 dpi. Although this timeframe covers the major replication and dissemination phases reported in previous JEV studies, extending the observation period to 21 days or beyond would allow better assessment of potential late-stage transmission in *Cx. pipiens pallens*, which may influence its epidemiological significance in urban settings. The relatively low viral titers observed in *Cx. pipiens pallens* may also have underestimated its actual vector competence. This underestimation could be attributed to the limited oral susceptibility of laboratory colonies, potential detection limits of the assays used, and the absence of environmental factors that may enhance viral replication and transmission under natural conditions. Nevertheless, our findings have important implications for JE epidemiology. The confirmed competence of *Cx. pipiens pallens* for GV—currently the dominant genotype in the ROK—underscores the need to expand vector surveillance efforts to include urban-adapted species. As JE incidence continues to rise in metropolitan areas, vector control and vaccination strategies must adapt to reflect the evolving transmission landscape.

## Conclusions

This study demonstrated distinct vector competence profiles of *Cx. tritaeniorhynchus* and *Cx. pipiens pallens* for JEV GIII and GV under controlled laboratory conditions. *Culex tritaeniorhynchus* consistently exhibited high IR, DR, and TR for both genotypes, confirming its role as the primary JEV vector in the ROK. In contrast, *Cx. pipiens pallens* showed lower overall susceptibility, with genotype-dependent variation suggesting limited replication and transmission efficiency. Despite these laboratory findings, the predominance of *Cx. pipiens pallens* in urban areas highlights its potential involvement as a secondary vector under changing ecological conditions. Continued surveillance encompassing diverse mosquito species and host-feeding analyses is essential to elucidate the transmission cycle of JEV GV and reinforce the need for integrated vector-host monitoring and ecological studies in both rural and urban environments.

## Data Availability

No datasets were generated or analysed during the current study.

## Abbreviations

CPE: Cytopathic effects
Ct: Cycle threshold
dpi: Days post-infection
DR: Dissemination rate
FBS: Fetal bovine serum
GIII: Genotype III
GV: Genotype V
IR: Infection rate
JE: Japanese encephalitis
JEV: Japanese encephalitis virus
KDCA: Korea Diseases Control and Prevention Agency
MEM: Minimal essential medium
NS: Non-structural protein
P/S: Penicillin/streptomycin
PBS: Phosphate-buffered saline
PFU: Plaque-forming units
PTR: Population transmission rate
ROK: Republic of Korea
TCID: Tissue culture infectious dose
TR: Transmission rate
